# Porcine Epidemic Diarrhea Virus RNA Present in Commercial Spray-Dried Porcine Plasma Is Not Infectious to Naïve Pigs

**DOI:** 10.1371/journal.pone.0104766

**Published:** 2014-08-12

**Authors:** Tanja Opriessnig, Chao-Ting Xiao, Priscilla F. Gerber, Jianqiang Zhang, Patrick G. Halbur

**Affiliations:** 1 The Roslin Institute and The Royal (Dick) School of Veterinary Studies, University of Edinburgh, Roslin, Midlothian, United Kingdom; 2 Department of Veterinary Diagnostic and Production Animal Medicine, College of Veterinary Medicine, Iowa State University, Ames, Iowa, United States of America; Tulane University, United States of America

## Abstract

Porcine epidemic diarrhea virus emerged in North America in April 2013 and has since been identified in 30 U.S. States, Canada and Mexico. The rapid spread of PEDV has raised concerns about the role of feed and particularly pork-by-product components such as spray-dried porcine plasma (SDPP) in PEDV transmission. The aim of this study was to determine the infectivity of PEDV RNA present in commercial SDPP. Specifically, 40 3-week-old PEDV naïve pigs were randomly assigned to one of five treatment groups. At day post inoculation (dpi) 0, NEG-CONTROL pigs were sham-inoculated, PEDV-CONTROL pigs received cell culture propagated PEDV, and SDPP-CONTROL pigs were switched to a diet with 5% SDPP containing 5.1±0.1 log_10_ PEDV RNA copies/g. To evaluate a potential positive effect of anti-PEDV antibodies in SDPP on PEDV challenge, four days prior to PEDV challenge the pigs in the SDPP-PEDV group were switched to and remained on a 5% SDPP diet through dpi 28. Another group, EGG-PEDV, was orally administered a commercial egg-derived liquid PEDV globulin product from dpi -4 through 6. All PEDV-CONTROL pigs began shedding PEDV in feces by dpi 3 and seroconverted between dpi 7 and 14, whereas pigs in NEG-CONTROL and SDPP-CONTROL groups remained PEDV RNA negative and did not seroconvert to PEDV for the study duration. This indicates no evidence of infectivity of the PEDV RNA in the SDPP lot utilized. Furthermore, under the study conditions SDPP or egg-derived liquid PEDV globulin addition did not significantly alter PEDV-shedding or overall disease course after experimental challenge.

## Introduction

Porcine epidemic diarrhea virus emerged in North America in April 2013 [1]. Since the initial discovery, PEDV has spread rapidly through the pig population and is present in 30 U.S. States, Canada and Mexico as of May 2014. PEDV, an *Alphacoronavirus*, is a member of the *Coronaviridae* family which is a group of single-stranded, positive-sense RNA viruses [2]. PEDV isolates can be divided into genogroups 1 and 2 [3].

Spray-dried porcine plasma (SDPP) is a common feed additive to nursery pig diets to promote growth and improve overall pig health [4]. The raw plasma utilized is commonly collected in slaughter house plants from healthy pigs, transported to spray drying facilities and immediately processed. While it is possible that the plasma contains trace amounts of viral DNA or RNA [5], experimental feed trials using a much higher than normal percentage of SDPP over prolonged periods of time have indicated no infectivity of common viruses such as porcine circovirus type 2 (PCV2) [5], [6]. Moreover, SDPP also contains high levels of neutralizing antibodies [7] which ultimately contribute to the biosafety of the final SDPP.

With the rapid spread of PEDV in North America, concerns over SDPP as a possible source of PEDV introduction into herds were raised and led to the recommendation to discontinue use in some countries such as the UK [8]. While PEDV as an RNA virus is unlikely to survive the commercial spray-drying process, controlled experimental studies are needed to further prove or disprove these transmission speculations as SDPP is an important component of nursery diets in many production systems.

Effective vaccines for PEDV are still urgently needed and the current lack of vaccines or other tools for prevention and control of PEDV in North America has forced producers to utilize alternative strategies such as avian derived immunoglobulins to attempt to mitigate the effects of PEDV. However, little information is available on the efficacy of these products to protect pigs against PEDV in the U.S. or elsewhere. Past studies also demonstrated that the use of SDPP improved average daily weight gain and decreased severity of enteric disease in piglets challenged with pathogenic *Escherichia coli* [9], [10]; therefore, a reduction of the negative effects associated with PEDV due to SDPP appeared reasonable. The objectives of this study were (1) to determine the infectivity of commercial SDPP confirmed positive for PEDV RNA and (2) to evaluate a potential protective effect of commercial SDPP and an egg-derived liquid PEDV globulin product.

## Materials and Methods

### Ethic statement

The experimental protocol was approved by the Iowa State University Institutional Animal Care and Use Committee (Approval No. 2-14-7742-S; approved on the 5^th^ of March 2014).

### Animals and housing

Forty, 2-week-old, colostrum-fed, crossbred piglets from a commercial herd free of PEDV and porcine reproductive and respiratory syndrome (PRRSV) were arbitrarily selected, transported to the Iowa State University Livestock Infectious Disease Isolation Facility, and housed in four separate biosecurity level 2 rooms. Three of the rooms contained a 3.8×1.5 m pen on a solid concrete floor, a self-feeder, and one nipple drinker. Two similar pens were placed at opposite ends of a larger 4^th^ room with 3.5 m separation between the two pens. Personnel handling the pigs were required to shower and change clothes, boots, gloves before entering each room. Prior to start of the experiment, all pigs were fed an age appropriate starter diet free of animal protein (produced on the 13^th^ of February 2014; Heartland Coop, Prairie City, Iowa, USA).

### Spray-dried porcine plasma (SDPP) production

The SDPP used for this study was from lot No. A4051110 (APC Inc., Ankeny, Iowa, USA) and was produced on the 20^th^ of February 2014. The inlet temperature in the spray drier was 204±5°C and the outlet temperature was 79±1.9°C. The moisture of the product during the run was 7.35±0.7%. This product was within normal production conditions and can be considered typical of commercial SDPP protein. Three different SDPP samples were collected; from the beginning, the middle, and at the end of the batch production cycle. The SDPP plasma lot used in this trial contained 5.1±0.1 log_10_ PEDV RNA copies/g and had IgG antibodies against PEDV at the ELISA optical density (OD) value of 0.45±0.0 at a dilution of 1∶10 in phosphate-buffered saline (PBS).

### Feed formulation

The selected commercial SDPP was incorporated into a non-pelleted, complete feed, at a final inclusion of 5%. Based on average intake of 215 g/d for a 28 day old pig fed for 28 d, average plasma consumption was 10.5 g/d, for a total consumption of 301 g of SDPP per pig. In addition, a diet without SDPP was also produced to provide equivalent dietary energy and lysine compared to the diet containing SDPP.

The SDPP diet contained 3.3±0.3 log_10_ PEDV RNA copies/g whereas the negative control diet was PEDV RT-PCR negative. After arrival at the research facility the bagged feed was stored in separate anterooms for each group at ambient room temperature (approximately 18°C). The interval from SDPP production to initiation of feeding the pigs in this trial at -4 dpi (20^th^ March 2014) for the SDPP-PEDV group or 0 dpi for the SDPP-CONTROL group (24^th^ March 2014) was between 28 and 32 days which can be considered typical in the U.S. and accounts for the time from production, transport to the feed mill, distribution to the farm, and administration to the pigs. Feed samples were obtained on a weekly basis from each anteroom and confirmed to (1) be PEDV negative (negative control feed) or (2) to contain 3.2±0.5 log_10_ PEDV genomic equivalent copies/g (SDPP positive feed ratio) without apparent reduction over time.

### Experimental design

After a one-week acclimation period, the pigs were randomly divided into five groups (Table 1) with eight pigs in each group including: Treatment 1 NEG-CONTROL (no SDPP in the feed; pigs not inoculated with PEDV), Treatment 2 PEDV-CONTROL (no SDPP in the feed; pigs inoculated with PEDV), Treatment 3 SDPP-CONTROL (5% commercial SDPP in the feed from dpi 0 to 28; pigs not inoculated with PEDV), Treatment 4 SDPP-PEDV (5% commercial SDPP in the feed from dpi -4 to 28; pigs inoculated with PEDV), and Treatment 5 EGG-PEDV (no SDPP in the feed; 5 ml per day orally from dpi -4 to 6 of EPFX/EGCO Protein's Farrow X1, Provanco Feeds, Le Center, Minnesota, USA; pigs inoculated with PEDV). The negative control diet used for treatments 1, 2 and 5 did not contain any blood-derived protein. All diets were formulated to meet the nutritional requirements of the pig [11] and were provided in self-feeders to assure ad libitum consumption. Treatment 2, 4 and 5 pigs were inoculated orally with 10 ml of a cell-culture propagated PEDV inoculum by slowly dripping the inoculum into the oral cavity of the pig. Blood samples were collected in serum separator tubes (Fisher Scientific, Pittsburgh, Pennsylvania, USA) at arrival, on the day of inoculation, and at dpi 3, 7, 14, 21 and 28, centrifuged at 2000×g for 10 min at 4°C, and tested for the presence of anti-PEDV-IgG antibodies and PEDV RNA. Similarly, fecal swabs were collected at arrival and dpi 0, 3, 5, 7, 14, 21 and 28 and tested for the presence of PEDV RNA. Specifically, the swabs were collected using polyester swabs (Fisher Scientific, Inc.) and stored in 5 ml plastic tubes (Fisher Scientific, Inc.) containing 1 ml of sterile saline solution (Fisher Scientific, Inc.). All pigs were weighed at -4 dpi and at dpi 28.

**Table 1:** Experimental design.

Group Designation	No. of pigs	Diet or treatment	Inoculation	Necropsy	
			dpi 0	dpi 3	dpi 28
NEG-CONTROL	8	No SDPP or other treatment	PBS	3	5
SDPP-CONTROL	8	5% SDPP^1^ (dpi 0 to dpi 28)	PBS	3	5
PEDV-CONTROL	8	No SDPP or other treatment	PEDV	3	5
SDPP-PEDV	8	5% SDPP (dpi -4 to dpi 28)	PEDV	3	5
EGG-PEDV	8	Ab^2^ treatment (dpi -4 to dpi 6)	PEDV	3	5

Abbreviations: dpi, day post inoculation; SDPP, spray-dried porcine plasma; PBS, phosphate-buffered saline; PEDV, porcine epidemic diarrhea virus.

1 Spray-dried porcine plasma from APC, Inc., Ankeny, Iowa, USA.

2 EPFX/EGCO Protein's Farrow X1, Provanco Feeds, Le Center, Minnesota, USA.

### Treatments

#### SDPP-free, PEDV-negative control feed

Pigs in the NEG-CONTROL, PEDV-CONTROL, and the EGG-PEDV groups were feed a diet free of SDPP and PEDV RNA from dpi 0 through 28.

#### SDPP (5%), PEDV-positive control feed

Pigs in the SDPP-PEDV group were fed the 5% SDPP diet from dpi -4 through 28 and pigs in the SDPP-CONTROL group received the SDPP diet from dpi 0 until 28.

#### Anti-PEDV globulin

A liquid egg protein formulation (EPFX/EGCO Protein's Farrow X1, Provanco Feeds, Le Center, Minnesota, USA; Lot# LS 60030614-1) was obtained from a commercial source and was administered daily in a 5 ml volume orally from dpi -4 to dpi 6. According to company specifications, the product was tested for anti-coronavirus antibodies and had an OD value of 1.0 based on incubating the liquid egg protein formulation diluted 1∶10 in PBS on ELISA plates coated with various coronavirus antigens. In that test, samples with OD values greater than 0.2 were considered positive.

### PEDV inoculation

PEDV-CONTROL, SDPP-PEDV and EGG-PEDV pigs were experimentally infected with 10 ml of the 5^th^ passage of the PEDV isolate 13-19338E [12] at a tissue culture infective dose (TCID_50_) of 5×10^2^ per ml via the oral route on dpi 0.

### Serology

Sera from blood samples collected at arrival and at dpi -4, 0, 7, 14, 21 and 28 were tested for the presence of anti-PEDV IgG antibodies by an *in-house* spike gene 1 (S1)-based indirect ELISA and by indirect immunofluorescence assay (IFA). Briefly for the S1-ELISA, an immunogenic fragment spanning amino acids 1 through 718 of the S1 domain of the PEDV IA1 strain [3] expressed in an eukaryotic expression vector was used as antigen. Microtiter plates were coated with the S1 polypeptide diluted in carbonate coating buffer and incubated overnight at 4°C. Plates were then blocked with 1% bovine serum albumin for 2 h at 22°C and incubated with samples diluted 1∶100 in PBS containing 10% goat serum for 30 min at 37°C. After a washing step, a 1∶20,000 diluted peroxidase-conjugated goat anti-swine IgG (Jackson ImmunoResearch) was added and incubated at 37°C for 30 min. The peroxidase reaction was visualized by using tetramethylbenzidine-hydrogen peroxide solution as the substrate for 10 min at room temperature and stopped by adding 50 µl of 2.5 M sulfuric acid to each well. For the S1-ELISA, samples with an OD value greater than 0.3 were considered positive, samples with OD values between 0.2 and 0.3 were considered as indeterminate, and samples with OD values below 0.2 were considered negative. Samples were tested in 2-fold dilutions with the IFA ranging from 1∶40 to 1∶320. Positive signals at a sample dilution of 1∶40 or higher were considered positive.

### RNA extraction

Total nucleic acids were extracted from serum samples, fecal swab suspensions and inoculation materials (raw plasma samples and reconstituted spray-dried porcine plasma powders) using the MagMax Pathogen RNA/DNA Kit (Applied Biosystems, Life Technologies, Carlsbad, California, USA) and an automated DNA/RNA extraction system (Thermo Scientific Kingfisher Flex, Thermo Fisher Scientific, Pittsburgh, Pennsylvania, USA) according to the instructions of the manufacturer.

### Quantitative real-time RT-PCR for PEDV

For development of the quantitative PEDV real-time RT-PCR assay 31 genomic sequences of PEDV were downloaded from GenBank and aligned with the Lasergene package (DNAStar Inc., Madison, Wisconsin, USA). A pair of PEDV detection primers (PEDVDF: 5′-GTGGCTCTCAAACTGTTTTACGTTG-3′; PEDVDR: 5′-GACACCACAATCTGAAGCACAACAC-3′) and a TaqMan probe (PEDVprob: 5′-6-FAM-ACGGCGTCCTATGCTTTGTACTAAGTGTG-BHQ-3′) were designed within the conservative ORF1b gene by Primer Express software (version 3.0; Applied Biosystems, Foster City, California, USA) to cover a region of 149 nucleotides. The probe was labeled with 6-Carboxyfluorescein (FAM) at the 5′ end and Black Hole Quencher (BHQ) at the 3′ end.

The real-time RT-PCR was carried out in 96-well plates, with each reaction consisting of a total volume of 25 µl, containing 12.5 µl TaqMan One-Step RT-PCR Master Mix Reagent (Applied Biosystems), 6 µl RNA, 0.625 µl 40× MultiScribe and RNase Inhibitor, 1 µl each of the two primers (10 µM), 0.5 µl probe (10 µM) and 3.375 µl RNase-free water. Amplification reactions were performed using an Applied Biosystems 7500 Fast Real-Time PCR System (Applied Biosystems) under universal conditions: 30 min at 50°C, 10 min at 95°C, followed by 40 cycles of 15 s at 95°C and 1 min at 60°C. A sample was considered negative if no cycle threshold (Ct) was detected during the 40 amplification cycles. PEDV genomic loads per fecal swab were calculated by multiplying the individual results from the quantitative real-time RT-PCR by 166.7 (50 µl RNA preparation eluted from 50 µl fecal swab suspension ×20 (1 ml saline per 50 µl elution buffer) per 6 µl PCR input).

To obtain standards, the PCR products with primers PEDVDF/DR were purified and cloned into pGEM-T Easy Vector System (Promega). The recombinant plasmids were transformed into TOP10 *Escherichia coli* competent cells (Invitrogen) and propagated following the instructions of the cloning kit manual. The plasmids were extracted using a QIAprep Spin Minipreps Kit (Qiagen) according to the manufacturer's instructions, quantified using a spectrophotometer (NanoPhotometer; Implen) and sequenced. The confirmed plasmids were used as standards in the real-time RT-PCR assay to determine assay sensitivity and to quantify viral loads in the fecal swabs. The standard curve and the sensitivity of the real-time RT-PCR were carried out and determined as described previously [13]. The real-time RT-PCR assay was used on 10-fold serial dilutions of the PEDV plasmid, with a detection limit of 5 genome equivalents which occurred around a Ct of 36. The determined slope, R2 and intercept value of the standard curve were −3.455, 0.99 and 38.3. The specificity of the probe was confirmed by BLAST analysis and by testing samples positive for other RNA viruses available in the laboratory, including PRRSV, transmissible gastroenteritis virus, porcine respiratory coronavirus, porcine deltacoronavirus, porcine astrovirus and swine hepatitis E virus. No cross-amplification was observed, indicating that this real-time RT-PCR assay allowed specific, efficient and sensitive detection of PEDV. All RNA extracts were tested for the presence of PEDV RNA by the quantitative real-time RT-PCR assay.

### Sequencing

To confirm that the PEDV from the experimentally infected pigs was the same as the virus in the inocula, the S1 gene and nucleocapsid gene were amplified and sequenced utilizing the following primers: PEDVS1F (5-TTCTAATCATTTGGTCAACGTAAAC-3), PEDVS1R (5-TACTCATACTAAAGTTGGTGGGAATAC-3), PEDVNF (5- GTGCTTCATTTAGTCTAAACAGAAAC-3) and PEDVNR (5-GACATTACCACTGGCTTACCGT-3). The PCR products were purified using the QIAquick PCR Purification Kit (Qiagen) according to the manufacturers' directions and sequenced at the Iowa State University DNA facility. The sequences were aligned with published data using BLAST at the National Center for Biotechnology Information (NCBI) (http://www.ncbi.nlm.nih.gov/) and compiled using Lasergene software and the Clustal W alignment algorithm (DNAStar, Madison, Wisconsin, USA).

### Necropsy

All pigs were humanely euthanized by pentobarbital overdose (Fatal-plus, Vortech Pharmaceuticals, LTD, Dearborn, Michigan, USA) and three pigs per group were necropsied at dpi 3 and the remaining five pigs were necropsied at dpi 28. Severity of macroscopic lesions in the small and large intestines and consistency of the colon content (normal, fluid, liquid) were estimated by a pathologist blinded to the treatment status. Six sections of small intestines, three sections of large intestines, and one section of lung were collected at necropsy and fixed in 10% neutral-buffered formalin and routinely processed for histological examination. In addition, intestinal content was collected from the cecum of each animal and stored in individual 50 ml plastic centrifuge tubes (Fisher Scientific, Inc.). Sections of small and large intestines were also collected fresh and stored at −80°C.

### Histopathology

Microscopic lesions were evaluated by a veterinary pathologist blinded to treatment status. Sections of small and large intestines were evaluated for the presence of inflammation, villus atrophy, and necrosis and scored from 0 (none) to 3 (severe). Lung sessions were evaluated for presence of interstitial pneumonia and bronchiolitis.

### PEDV immunohistochemistry (IHC)

PEDV-specific antigen was detected by immunohistochemistry (IHC) on selected formalin-fixed and paraffin-embedded sections of intestinal sections using monoclonal antibody specific for PEDV (BioNote, Hwaseong-si, Gyeonggi-do, Korea) as described [1], [14]. The amount of PEDV antigen was scored by a pathologist blinded to treatment status. Scores ranged from 0 (no signal) to 3 (abundant, diffuse staining).

### Statistical analysis

For data analysis, JMP software version 10.0.2 (SAS Institute, Cary, North Carolina, USA) was used. Summary statistics were calculated for all the groups to assess the overall quality of the data set including normality. Statistical analysis of the data was performed by one-way analysis of variance (ANOVA) for continuous data (log_10_ transformed RT-PCR data, ELISA data, and average daily weight gain). A p-value of less than 0.05 was set as the statistically significant level. Pairwise test using Tukey's adjustment was subsequently performed to determine which differences among groups were statistically significant. Non-repeated nominal data (histopathology scores and IHC scores) were assessed using a non-parametric Kruskal-Wallis one-way ANOVA, and if there was a significant difference, pairwise Wilcoxon tests were used to evaluate differences among groups. Differences in prevalence were determined by using chi-square tests.

## Results

### Clinical presentation

The average daily weight gain is summarized in Table 2. There were no significant differences among groups. Clinical signs were characterized by development of mild diarrhea defined by semi-solid to fluid feces around dpi 2 in PEDV-infected groups. By dpi 4, PEDV-infected pigs were lethargic and had moderate amounts of liquid greyish diarrhea. Between dpi 6 to 9 the degree of clinical signs appeared less severe in SDPP-PEDV pigs compared to EGG-PEDV and PEDV-CONTROL. Clinical signs between dpi 10 to 11 and feces composition returned to normal standards. There were no remarkable clinical signs in any of the NEG-CONTROL pigs or SDPP-CONTROL pigs.

**Table 2:** Average daily gain in grams (±SEM) as determined on all pigs that were kept until 28 days post inoculation.

Group designation	No. of pigs	Average daily gain from dpi -4 to 28
NEG-CONTROL	5	381.3±41.9
SDPP-CONTROL	5	446.6±39.9
**PEDV-CONTROL**	**5**	**358±53.0**
**SDPP-PEDV**	**5**	**360±19.0**
**EGG-PEDV**	**5**	**348.6±45.8**

Bold areas indicate the pigs experimentally inoculated with PEDV

### Anti-PEDV antibody levels

**Figure 1:**
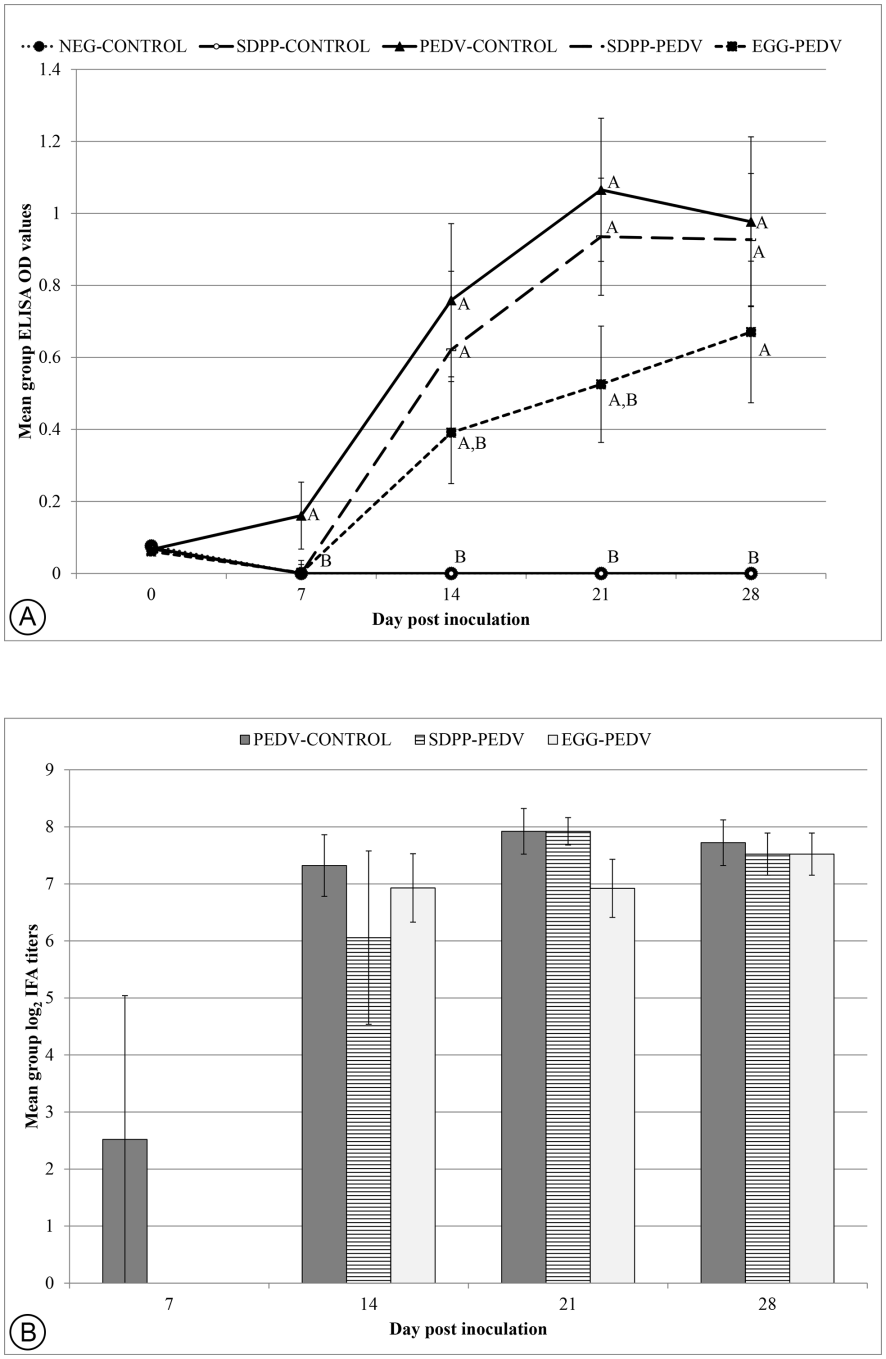
Group mean antibody response in serum over time. (**A**) Group mean S1 ELISA OD values ±SEM. An OD value greater than 0.3 was considered positive, samples with OD values between 0.2 and 0.3 were considered suspect, and samples with OD values less than 0.2 were considered negative. (**B**) Group mean IFA titers (±SEM). Samples were tested in 2-fold dilutions with the IFA ranging from 1∶40 to 1∶320. Positive signals at a sample dilution of 1∶40 or higher were considered positive. The data were analyzed by one-way ANOVA method followed by Tukey's pairwise test using the JMP software version 10.0.2 (SAS Institute, Cary, North Carolina, USA). Different superscripts (^A,B^) indicate significant different group means on selected days (p<0.05).

Classification of individual pigs as antibody positive or negative was identical for the S1-ELISA and the IFA. Antibodies against PEDV were initially detected by dpi 7 in the PEDV-CONTROL pigs (2/8) (Fig. 1). By dpi 14, 100% (5/5) of PEDV-CONTROL pigs, 80% (4/5) of the SDPP-PEDV pigs and 60% (3/5) of the EGG-PEDV pigs had seroconverted to PEDV. At dpi 21 and 28, all PEDV-infected pigs were seropositive. Using the S1-ELISA, EGG-PEDV pigs had lower anti-PEDV OD values compared to PEDV-CONTROL and SDPP-PEDV pigs at dpi 14, 21 and 28; however, the difference was not significant (Fig. 1A). PEDV-antibodies were not detected in any of the NEG-CONTROL pigs or the SDPP-CONTROL pigs throughout the study.

### PEDV shedding in feces

**Figure 2:**
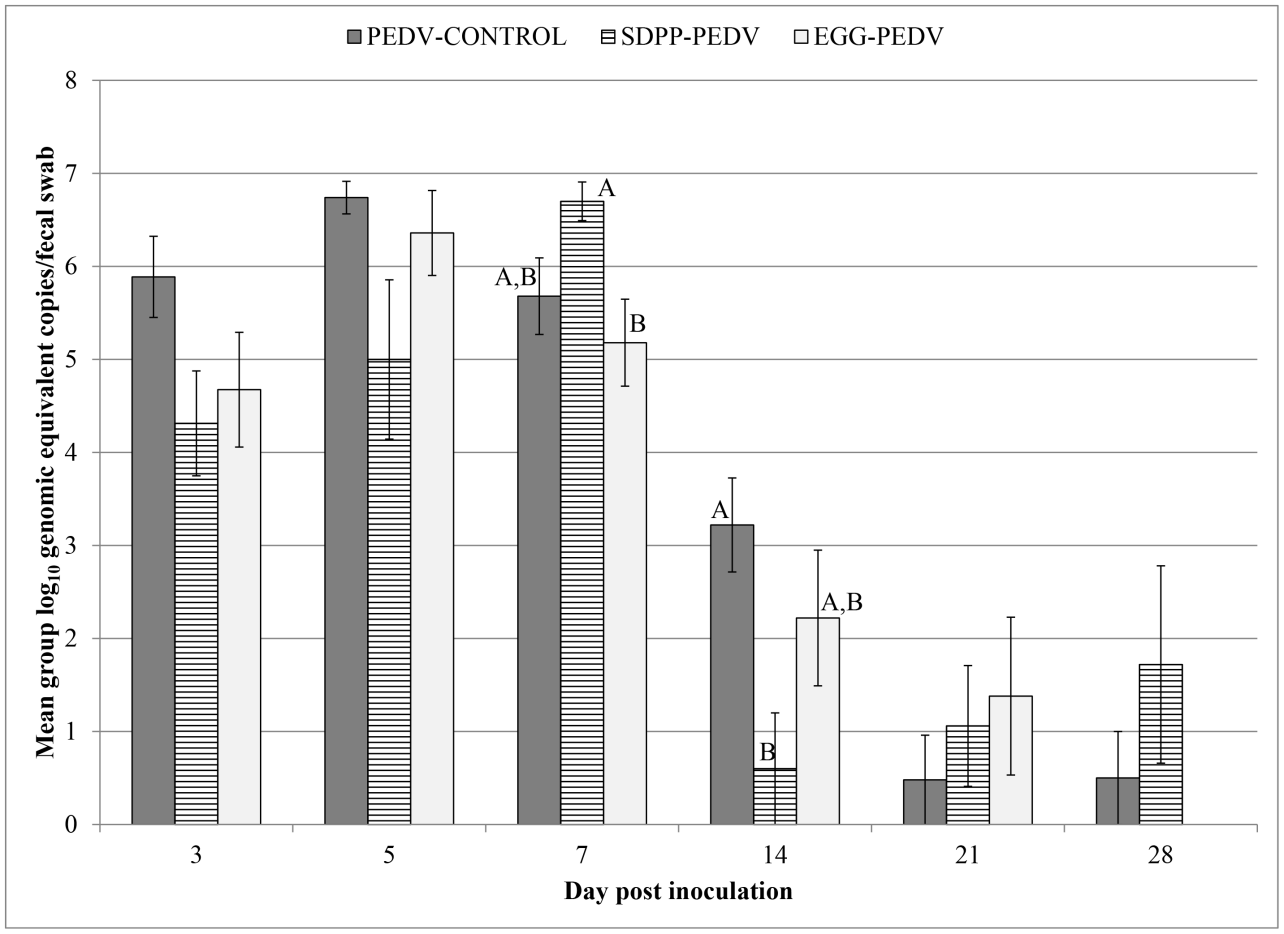
Group mean PEDV RNA levels in fecal samples over time. Group mean log_10_ PEDV RNA levels (±SEM) were determined in fecal samples from PEDV-infected pigs (PEDV-CONTROL, SDPP-PEDV, EGG-PEDV) collected from day post inoculation (dpi) 3 through 28. The data were analyzed by one-way ANOVA method followed by Tukey's pairwise test using the JMP software version 10.0.2 (SAS Institute, Cary, North Carolina, USA). Different superscripts (^A,B^) indicate significant different group means on selected days (p<0.05).

PEDV RNA was not detected in any of the NEG-CONTROL or SDPP-CONTROL pigs throughout the study (Table 3). PEDV RNA was detected in fecal swabs of all PEDV-infected groups at dpi 3, 5 and 7. By dpi 14, fewer pigs were shedding PEDV RNA in the SDPP-PEDV (1/5) and EGG-PEDV (4/5) groups. At dpi 28, the only PEDV inoculated group that did not contain PEDV RNA in feces was the EGG-PEDV group. Prevalence and mean group amount of PEDV RNA in feces are summarized in Table 3 and Fig. 2. Further sequencing of PCR positive samples confirmed 100% identity of the PEDV compared to the strain used for inoculation.

**Table 3:** Prevalence of PEDV RNA in fecal swabs at different days post PEDV inoculation.

Group	0	3	5	7	14	21	28
NEG-CONTROL	0/8	0/8	0/5	0/5	0/5	0/5	0/5
SDPP-CONTROL	0/8	0/8	0/5	0/5	0/5	0/5	0/5
**PEDV-CONTROL**	**0/8**	**8/8**	**5/5**	**5/5**	**5/5**	**1/5**	**1/5**
**SDPP-PEDV**	**0/8**	**8/8**	**5/5**	**5/5**	**1/5**	**2/5**	**2/5**
**EGG-PEDV**	**0/8**	**8/8**	**5/5**	**5/5**	**4/5**	**2/5**	**0/5**

Bold areas indicate the pigs experimentally inoculated with PEDV.

### PEDV viremia

PEDV RNA was detected in low amounts in individual PEDV-infected pigs at dpi 3 and 7 but not at dpi 14, 21, or 28. Specifically, 2/8 PEDV-CONTROL pigs, 1/8 SDPP-PEDV pigs and 4/8 EGG-PEDV pigs were positive for PEDV RNA on dpi 3, and 1/5 positive controls, 2/5 SDPP-PEDV pigs and 1/5 EGG-PEDV pigs were RT-PCR positive on dpi 7. The PEDV load in positive serum samples ranged from 2.2 to 3.2 log_10_ genomic equivalent copies per ml. PEDV RNA was not detected in any of the serum samples obtained from the NEG-CONTROL pigs or the SDPP-CONTROL pigs.

### Macroscopic lesions

By dpi 3, gross lesions were limited to thin-walled small intestines containing fluid grey-green intestinal content in PEDV-infected pigs (Table 4). By dpi 28, no remarkable macroscopic lesions were observed and the intestinal content appeared normal.

**Table 4:** Macroscopic appearance of the intestinal content, prevalence of PEDV antigen as determined by IHC and microscopic lesions in pigs euthanized at 3 days post inoculation.

Group designation	No. of pigs	Appearance of the intestinal content			PEDV IHC	Microscopic lesions		
						Villus Atrophy	Colitis	Presence of adherent *E. coli*
		Normal	Semi-solid	Fluid				
NEG-CONTROL	3	1	2	0	0/3 (0)^1^	0/3	0/3	0/3
SDPP-CONTROL	3	3	0	0	0/3 (0)	0/3	0/3	0/3
**PEDV-CONTROL**	**3**	**0**	**0**	**3**	**3/3 (3)**	**3/3 (3)**	**1/3 (1)**	**2/3**
**SDPP-PEDV**	**3**	**2**	**0**	**1**	**2/3 (1.5)**	**2/3 (1.7)**	**0/3**	**0/3**
**EGG-PEDV**	**3**	**0**	**1**	**2**	**1/3 (0.3)**	**2/3 (1.3)**	**2/3 (0.7)**	**2/3**

Bold areas indicate the pigs experimentally inoculated with PEDV.

1 Number of pigs affected/total number of pigs (group mean score).

### Microscopic lesions and PEDV antigen in tissues

**Figure 3:**
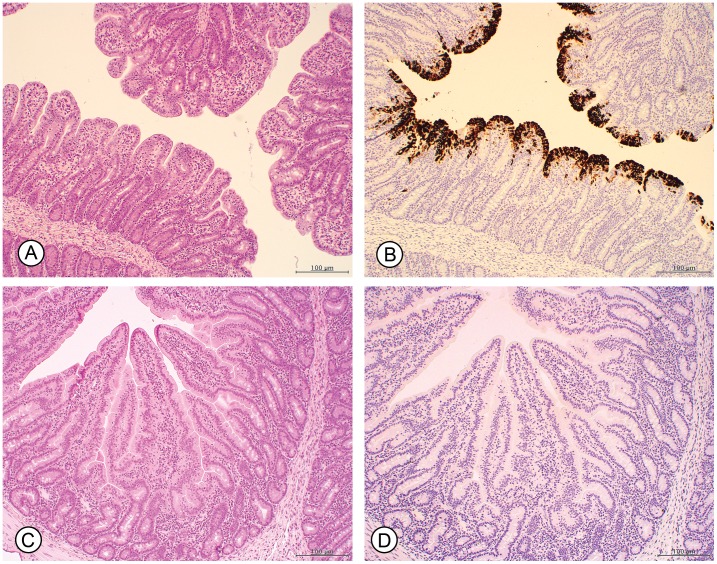
Microscopic lesions in the small intestines at 3 days post PEDV inoculation. (**A**) PEDV-CONTROL. HE. There is severe diffuse atrophy and fusion of villi of the small intestine. (**B**) PEDV-CONTROL. PEDV IHC. The majority of the enterocytes contain abundant PEDV antigen as indicated by brown staining of enterocytes. (**C**) SDPP-CONTROL. HE. The intestinal mucosa is normal and the villi are of normal length. (**D**) SDPP-CONTROL. PEDV IHC. PEDV antigen is not present.

By dpi 3, the majority of the PEDV-infected pigs had segmental, mild to severe villous atrophy and fusion in the small intestines associated with abundant PEDV antigen in the enterocytes along the tip and sides of the villi (Fig. 3). PEDV antigen staining was also observed in isolated cells in the lamina propria and Peyer's patches. Staining was not observed in the crypts of the small intestines or in any cells of the colon. Individual pigs had very mild multifocal necrosuppurative colitis associated with attaching and effacing *E. coli* (Table 4). The prevalence of PEDV antigen as determined by IHC stains in the PEDV-infected pigs is summarized in Table 4. PEDV antigen was not detected in any of the NEG-CONTROL pigs or SDPP-CONTROL pigs (Fig. 3).

By dpi 28, no microscopic lesions were observed in intestinal sections and PEDV antigen was not detected in any of the groups.

## Discussion

The emergence of PEDV in North America in April 2013 and the apparent inability to prevent the virus from spreading rapidly through the swine population has raised important questions on transmission between farms and countries. Concerns over the role of SDPP in PEDV transmission were raised, when geographically unrelated pig operations without known contact points, but with a common commercial feed source almost simultaneously became infected with PEDV [15], [16]. SDPP is derived from slaughter age pigs without clinical signs of disease. Recently it has been determined that under extreme experimental conditions, PCV2, a small, non-enveloped DNA virus that is very resistant to temperature changes, can survive the spray-drying process and retain infectivity [17]; however, the PCV2 in commercial product has been repeatedly demonstrated to not be infective [5], [6], [18]. Under similar conditions, RNA viruses like porcine reproductive and respiratory syndrome virus (PRRSV) were inactivated successfully [19]. The main objective of this study was to determine the infectivity of commercial SDPP positive for PEDV RNA in a conventional pig model.

In this study, PEDV-negative pigs were divided into five groups and four rooms and fed either a diet containing 5% SDPP confirmed positive for PEDV RNA or a control diet. One group was experimentally infected with PEDV by the oral route and served as positive control. Under the study conditions, none of the SDPP-CONTROL pigs developed clinical signs consistent with PEDV-infection, they did not shed PEDV RNA in feces and they did not seroconvert to PEDV indicating that the PEDV in the SDPP was not infectious. In contrast, the PEDV-CONTROL pigs had diarrhea from dpi 2 through dpi 10, shed PEDV RNA in feces from dpi 3 through 28, and seroconverted to PEDV.

Porcine plasma is obtained from blood collected in abattoirs. Presence of a virus in plasma is typically associated with an ongoing viremia. Previously, PEDV viremia has been reported in gnotobiotic pigs experimentally infected with PEDV genogroup 2 [20]. Similarly, in the present study PEDV RNA was detected in serum at the peak of disease, but viremia was short and of low magnitude (low numbers of PEDV genomic copies in real-time RT-PCR positive pigs at single time points). Therefore it appears unlikely that PEDV viremia and utilization of blood from viremic pigs is a main source of PEDV contamination of SDPP. Another potential source of PEDV RNA in raw blood may be attributed to contamination from swine carcasses during the blood withdrawal process. Sources of secondary contamination of the SDPP or swine feed throughout the distribution chain should be further investigated to reduce or prevent feed-associated transmission of PEDV.

Due to the lack of effective intervention tools against PEDV in North America, alternative methods are being investigated. Chicken egg antibodies have been used for prophylaxis and therapy of infectious disease in pigs for some time and have been suggested as a viable alternative to commonly used antimicrobial therapy [21]. Oral administration of IgY has been demonstrated to be cost effective and convenient [22]. Studies with *Escherichia coli* K88 [23] or F18 [24] indicated performance improvements and inhibition of bacterial shedding in treated pigs compared to untreated controls. Chicken egg yolk globulin against PEDV was found to reduce mortality in piglets after experimental PEDV challenge (2–3 ml were administered orally three times for one day) and also significantly improved survival rates of piglets during a field study in Korea [25]. In order to mimic what is done in the U.S. field, the liquid egg protein formulation (Farrow X1) obtained from hens immunized against PEDV and transmissible gastroenteritis virus (TGEV) was administered orally for 10 consecutive days to the EGG-PEDV group starting at 4 days prior to PEDV challenge. Due to presence of anti-chicken IgY antibody rather than pig IgG, Farrow X1 was not tested with the *in house* IgG ELISA, as results would not have been comparable due to using a different conjugate. However, based on company specifications, anti-coronavirus antibodies in high levels were present in the product. There was no significant clinical improvement and except for dpi 14 there was no significant reduction in PEDV shedding in treated (EGG-PEDV) versus untreated pigs (PEDV-CONTROL) although there was a numerical difference in the early phase of infection (dpi 3 and dpi 5). Reasons for this may include pathogen type, challenge route and dose, and timing/dosing of the EGG-PEDV treatment under the study conditions. Of note, PEDV shedding in this study was evaluated by RT-PCR which cannot distinguish between virus fragments or live virus. It is therefore unknown if the treatment would have affected transmission rates to naïve pigs and the overall environmental virus load.

Another potential way to neutralize infectious viruses present in the intestinal lumen is by feeding SDPP containing high levels of immunoglobulins [26]–[28]. It has also been determined that the dietary inclusion of SDPP enhanced the intestinal barrier function and reduced inflammation and subsequent presence of diarrhea in weaned pigs [26]. Some of the benefits were attributed to promotion of the intestinal development, increased antioxidant capacity and decreased production of inflammatory factors in the intestinal mucosa [29]. Interestingly, during acute PEDV infection at 3 dpi, adherent *E. coli* was not observed in any of the SDPP-PEDV pigs but was present in 2/3 EGG-PEDV pigs and in 2/3 PEDV-CONTROL pigs (Table 4). In addition, at dpi 14 there was significantly reduced PEDV shedding in the SDPP-PEDV group versus the EGG-PEDV group.

In this study, the SDPP-PEDV pigs were given the SDPP diet for 32 consecutive days starting at dpi -4 until dpi 28. This is longer than SDPP is typically fed in the field; however, it was done in this experiment to mimic the SDPP-CONTROL group which received the SDPP diet from challenge to dpi 28. Similar to the results for the EGG-PEDV group, the kinetics of PEDV infection was essentially no different in the SDPP-PEDV group compared to PEDV-CONTROL pigs except that the SDPP group appeared more active during the acute PEDV disease stage with less pronounced diarrhea as determined by several independent observers. It has been determined that apparent and standardized ileal digestibility of egg derived amino acids is lower when compared to SDPP [30] and a higher amino acid digestion could have contributed to the milder clinical signs in the present study. However, larger studies to evaluate this further are needed.

Under the conditions of this study, which were designed to mimic field conditions, a commercial PEDV positive SDPP product was not capable of transmitting PEDV to pigs. However, in contrast to anecdotal reports from the field, SDPP or anti-PEDV globulin addition did not significantly alter PEDV disease course or PEDV shedding after experimental challenge. However, fecal PEDV shedding in treated pigs (EGG-PEDV and SDPP-PEDV) was about 1 log lower compared to the POSITIVE-CONTROL pigs in the early stage of infection which could contribute to lower environmental PEDV loads and lower transmission rates to uninfected contact pigs.
